# Mitigation of Social Impacts of Alternative Road Development Plans Based on Local Wisdom in West Sumatra, Indonesia

**DOI:** 10.1155/2022/6895084

**Published:** 2022-12-05

**Authors:** Priyambodo, Kristian Buditiawan, Agung Budi Irawan, Dendy Setyawan, Herrukmi Septa Rinawati, Afriyanni, Momon, Elsa Yolarita, Vivi Ukhwatul Khasanah Masbiran, Imbang Dananjaya

**Affiliations:** ^1^Research and Development Agency of East Java Province, Surabaya, Indonesia; ^2^Research and Development Agency of Pekanbaru City, Jalan Abdul Rahman Hamid-Tenayan Raya Office Complex, Pekanbaru, Indonesia; ^3^Research and Development Agency of West Sumatra Province, Jalan Jenderal Sudirman No. 51, Padang, Indonesia; ^4^Surabaya Shipping Polytechnic, Surabaya, East Java, Indonesia

## Abstract

Infrastructure development, especially roads, is hampered by the fact of rejection based on urgency and attachment to the Ulayat land of the local community. Therefore, further models are needed in the form of mitigation based on culture and local wisdom to avoid conflicts and land disputes and changes in their use. This study aims to identify the social impacts of the bypass Amor Road construction and its mitigation strategies based on the perceptions of the community and stakeholders. This research study uses a descriptive qualitative research method. Data collection techniques were carried out through documentation studies, observation, and in-depth interviews. The data analysis technique was carried out using SIA (social impact analysis). SIA helps the community, government, and private sector to better understand and anticipate possible social problems and the changes they bring about as a result of the construction of the bypass Amor road. The results of the study conclude that the success of road construction depends on the government's mitigation strategy in adopting local values that live in the community which is then followed by the existence of a foundation and expansion of the fulfilment of community interests. This study recommends three things, namely, (1) conduct intensive and responsive socialization and approach; (2) approach community involvement through a community-based approach and traditional leaders; and (3) prepare alternative livelihoods based on community interest, local potential, market opportunities, and financial feasibility as well as providing capital for businesses.

## 1. Introduction

The problem of congestion occurs in West Sumatra Province which incidentally is not a row of densely populated provinces in Indonesia (Indonesian Central Statistics Agency). This congestion occurs specifically on the Padang to Bukittinggi national road section, which is the main access to several areas inside and outside the province of West Sumatra. Some of the areas that this route will pass include Padang Panjang city, Tanah Datar regency, Bukittinggi city, Pasaman regency, Payakumbuh city, Fifty Cities regency to North Sumatra, and Riau Province. Besides being caused by an increase in the volume of vehicles that is not balanced with the expansion of roads, it is also caused by the intensity of other activities such as infrastructure development along the route, accidents, transportation mobility during holidays, disasters, and religious moments. This condition then made a commitment to formulate a road infrastructure development plan on this route considering the high urgency in overcoming congestion. The best option for the construction of the road is through the Bypass Road Extension Development plan to Amor. Road construction in the area, apart from overcoming congestion, also has the opportunity to reactivate the Amor market which is the economic driver of the local community and is traversed by the path in the road construction plan.

Although efforts to overcome congestion in West Sumatra have been carried out through a road construction plan and are in the consolidation stage, the road construction plan is experiencing problems due to the weak support and legitimacy of the local community. These constraints are particularly in the context of land acquisition to build road infrastructure, land/asset ownership status, and the low level of support from the community regarding the urgency and attachment of the local community's Ulayat lands. The paradox that occurs is a form of development dilemma, namely, a solution to overcome congestion (public interest) which is hindered by the fact of rejection on the basis of the urgency and attachment of the Ulayat land to the local community (local custom). The government itself has taken various approaches to development at the consolidation stage so that a further model is needed in the form of mitigation based on culture and local wisdom to avoid conflicts and disputes over land and changes in its use. In other words, the root of the development problem occurs due to a clash of structural policies with the cultural context of the development area related to the traditional heritage of the local community in West Sumatra.

Several studies have basically shown that road infrastructure development creates various positive and negative impacts on the socioeconomic sphere [1–3], to impacts on development. Sustainability and the environment [4–6]. In the socioeconomic context, Gibbons et al. [1] explain that the economic impact lies in the benefits of the road network built on local labor productivity variables and wages when using transportation access patterns and achieving time efficiency. Case study research conducted by Khanani et al. [2] has a similar opinion regarding the economic impact in the business sector with Gibbons et al. [1]; as well as increasing job opportunities, volume density and housing development (gentrification), the value of buying and selling and renting houses, land value. Lee [7] finds that there is a key role of transport infrastructure investment in strengthening the level of agglomeration and the resulting economic benefits for production and economic actors, by suggesting policies for transport projects to be implemented to maximize cost-effectiveness and to ensure that agglomeration is promoted in development areas.

On the other hand, there is a negative impact from the existence of infrastructure development projects that trigger social conflicts as the findings of Khanani et al. [2] explain the negative impact of road infrastructure development in the form of shifting the places of the poor to the interior which also changes the social pattern of the region that has been well organized, and also it is detrimental to the poor who live in the area of road infrastructure development and to the spatial impact of changes in agricultural land. This negative impact is also reinforced by survey research by Santos et al. [5] who explain the existence of public rejection on the basis of environmental impacts in the form of a decrease in air quality during infrastructure development. Several previous studies generally show that there are pros and cons related to the impact of infrastructure development, or in other words, there is no visible mitigation effort to prevent negative impacts so as to balance the impact of infrastructure development on the community. Thus, the research study seeks to examine this as the main form of novelty.

This study aims to identify the social impact of the bypass Amor road extension development and its mitigation strategy based on the perception of the community and stakeholders. The social impact mitigation strategy formulated in this study elaborates on the institutional role with local cultural values that live and are maintained in a society that adheres to Minangkabau traditional values. It is important to perform this research before development is carried out so that social problems of the community do not become obstacles that will hinder development later. It is hoped that this mitigation strategy can be used as a guideline by relevant stakeholders in carrying out the construction of the bypass Pasar Amor East Ring Road. In addition, this research study can also be a reference regarding the social picture of the community and the acquisition of customary land for infrastructure development in West Sumatra.

SIA is a decision-making method that is widely used by governments and organizations to assess the social impacts caused by planning, policies, and projects implemented [8, 9]. SIA offers sustainable planning by involving the community and considering the continuity of the relationship between the community and its social environment at each stage of project implementation so that it is expected to reduce conflicts that often occur in the project implementation process [10].

For Minang people, land not only has economic value but more than that land is the inheritance of hereditary ancestors, a symbol of tribal or people's honor. Therefore, improper methods often lead to rejection and vertical and horizontal conflicts in society. The SIA method allows us to formulate an appropriate social impact mitigation plan [11].

## 2. Method

### 2.1. General Approach

This study is in the form of answering two objectives, namely, first, to investigate the mitigation efforts carried out in line with the ongoing infrastructure development, and second, to investigate the suitability of mitigation efforts with the sociological conditions and customs of the communities affected by road construction. The design of the descriptive qualitative approach was chosen as the design of a specific, in-depth, and transparent approach from the research sources.

### 2.2. Interview and Field Data

The research location is in Agam Regency by applying interviews and field data studies to the local government regarding the ring road development plan. Interviews were applied to community leaders as well as public officials representing the Development Planning and Research Development Agency, Public Works Service, Community Empowerment Service, Cooperative Trade Industry Service and Small and Medium Enterprises, Banuhampu District Government, Sungai Pua District Government, and indigenous peoples (Nagari Ladang Laweh, Nagari Cingkariang, Nagari BatuPalano, Nagari Batagak, Nagari Sariak, Nagari Sungai Pua, Nagari Padang Lua, and Nagari Taluk). Field data retrieval in the form of documents in the form of road construction plans, Agam Regency Regional Spatial Plan (RTRW) documents, Agam Regency Medium Term Development Plan (RPJMD) documents and reports and documents and articles related to By Pass road construction.

### 2.3. Data Analysis and Processing

NVivo is a tool to assist researchers in conducting data management and data analysis processes systematically and rigorously [12]. Encoding based on characters is a feature of NVivo that is crucial for managing qualitative data, particularly when performing data analyses such as discourse, discussion, and literature reviews [13]. However, the coding carried out is influenced by the researcher's interpretation of the analyzed data [14]. NVivo helps us code and categorize the text in interview transcripts and other text data.

We use the help of the NVivo 12 Plus application to analyze data related to social impact analysis (SIA). The stages of data collection and analysis are carried out as follows:

- Secondary data collection and supporting documents (community characteristics, human resources, institutions, finance, infrastructure, and information technology)
- Identification of the involvement of stakeholders who will be involved in the ring road development plan
- Identification of social factors/variables, perceptions, and social conditions of the community through interviews
- Conduct of data analysis and determination of priority of activities
- Conduct of consultations with stakeholders and creation of a mitigation plan that ensures the involvement of the community and relevant stakeholders

## 3. Results

### 3.1. Characteristics of Infrastructure Development Areas

The construction of the East Ring Road bypass Pasar Amor crosses 2 subdistricts, namely, Sungai Pua District and Banuhampu District. Each subdistrict in which there is a village or in Minangkaubau custom, it is called a Nagari, where in each Nagari there is a traditional figure who acts as a representative in decision-making. In Sungai Pua subdistrict, there are 4 Nagari affected by the development impact from a total of 5 Nagari including BatuPalano, Sariak, Batagak, and Sungai Pua. Meanwhile, in Banuhampu District, there are 3 out of 7 Nagari affected by road infrastructure development, including LadangLaweh, Cingkariang, and Padang Lua. Details of the areas affected by the construction of infrastructure roads are described in Table 1.

In both Sungai Pua and Banuhampu subdistricts, the people in the two subdistricts have the potential for development in the agricultural, trade, services, and weaving sectors. In the agricultural sector, the community plays a role both as landowners and processors of agricultural products (included in the supply chain from planting to distribution) as well as labor (labor) in managing agricultural production. The link between the potential of the region and the road infrastructure development process is that the road infrastructure will later play a role in encouraging the local economy through the availability of distribution facilities and marketing of local products to cutting distribution channels which are actually much more efficient. This condition has the opportunity to confirm the findings of research arguments in China that road infrastructure is able to stimulate market economic growth [15] as well as the opinion of [16] which makes the public at large the emphasis on obtaining efficiency gains from road infrastructure development.

### 3.2. The Bypass Amor Road-Building Paradox: Urgency and Conflict

The early history of bypass road construction began in the late 1990s and is still continuing today. The road construction was stopped due to conflict and resistance from the community's rejection of the land consolidation process. The By Pass Road development plan is a part of the Padang- Bukittinggi-Payakumbuh-Riau Boundary Road (230 km) which is contained in the Long Term Development Plan (RPJP) document of West Sumatra Province 2005–2025. The initial plan for the construction of this bypass road was started from the Gaduik Ambacang-Pasar Amor Intersection which passed through the city of Bukittinggi and the regency of Agam.

The construction of the bypass road that passes through the city of Bukittinggi and Kabupaten Agam takes a long time. Road construction is constrained by land acquisition problems. Although most of the people of Bukittinggi city supported it, the land acquisition process for the construction of the bypass road was also marked by conflict and resistance from some other communities, especially regarding the method of land acquisition through consolidation [17].

Septiawan [18] found that the emergence of disputes in the land consolidation process for the construction of bypass road in Bukittinggi city was caused by internal and external factors. Internal factors occurred due to administrative errors and the continued impact of the previous road construction, while external factors were caused by a lack of community knowledge about land consolidation and violations of agreements by the community. The peak occurred when the bypass road was blocked in 2003 due to the dissatisfaction of one of the groups with the compensation provided by the government [19]. The land acquisition was only completed after the local government intervened and took an intensive and familial approach to the community in the context of land acquisition [18].

In several towns and districts, a land acquisition issue continues to impede infrastructure development. The majority of the land is owned by the community or tribal groups, and its usage by the local government requires protracted and difficult negotiations. Lack of access to the land causes certain infrastructure projects to fail. Most of all infrastructure development issues are related to land acquisition, and this problem continues to be the greatest one. Land acquisition might be a continuing challenge until the project infrastructure is put into action. Construction projects for infrastructure are plagued by social unrest and security issues (social conflict) (hereinafter referred to as construction projects). To ensure that Indonesian society develops fairly, this social dispute may prevent or even end all construction works [20].

This effort paid off with the release of all problematic land parcels [21]. Almost the same problem also occurred in the construction process of the bypass road in Agam Regency (Nagari Taluak and Padang Laweh). Landowners do not want their land to be acquired by way of consolidation but by way of market prices. The conflict was marked by ambushes by the community using sharp weapons on officers from the local government who would take measurements. Finally, the construction of the bypass road was stopped until Nagari Ladang Laweh.

The problem of land acquisition for development occurs a lot, especially for people who adhere to certain traditional and cultural values such as Minangkabau. Land has an important value in traditional community life which is passed down from generation to generation so that land acquisition must also go through certain mechanisms. The following are the results of an interview with the Nagari Ladang Laweh community leader.

“For the Bukittinggi people, they want to consolidate, but there are land owners who do not want to consolidate until Mr. Mayor Nurmatias is abandoned at this time. After several meetings and field surveys, there are quite a lot of problems regarding land, agriculture, buildings, and so on, plus all land cannot be certified. If you build it, it is aponyo, if it is a nephew, please even though it is not certified. It is quite heavy, but for us, there is no other solution.” (Interview with Y, 2020).

The road lanes in the Padang Luar market area from year to year are increasingly congested with passenger and goods traffic, especially during holidays and market days so that travel times become longer and cause congestion. The government has made efforts to reduce congestion in the Padang Lua market area by constructing a flyover and planning for market development backwards, such as at the Padang Baru market. These efforts faced many obstacles. The flyover development plan was rejected by the people of Padang Lua, Ladang Laweh, and Cingkariang because it was considered to have killed the businesses of the people who were under the flyover road. Furthermore, the development of the Padang Lua market backwards was constrained by land acquisition issues, land ownership issues, and the boundary issue of Nagari Padang Lua and Nagari Ladang Laweh.

**Figure 1:**
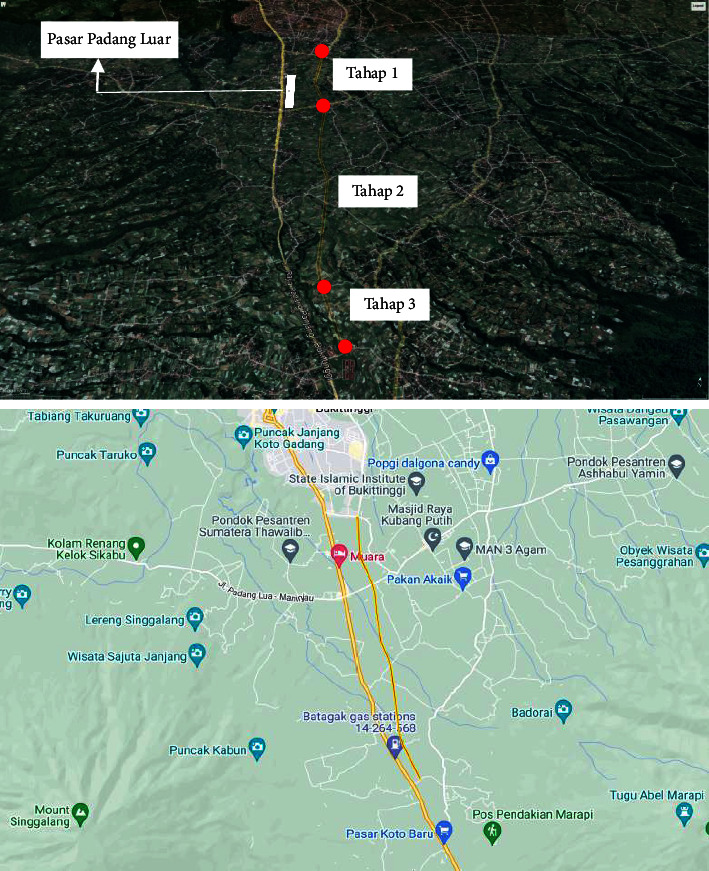
Location of affected areas.

The bypass road extension from Taluak to Amor market is considered as an alternative solution to solve the congestion problem at Padang Lua market. In addition, the road to Amor market is expected to revive economic activity in Amor market and the surrounding area. The discourse on the construction of the bypass road extension has been the discourse of the provincial and district governments several times. Thus, we are following up on the directive new route of the road to be built because the old route was not possible to continue (Table 2 and Figure 1).

### 3.3. Minangkabau Land and Customs

For communities affected by road infrastructure development who are the Minangkabau tribe, land is a component of customary rights for local communities and the existence of national regulations is strengthened in the form of the 1945 Constitution of the Republic of Indonesia (UUD 1945). Article 18B paragraph (2) of the 1945 Constitution states that the state recognizes and respects customary law community units and their traditional rights as long as they are still alive and in accordance with community development and the principles of the Unitary State of the Republic of Indonesia. Ulayat land is a form of traditional rights in the form of inheritance that is controlled communally (tribe, clan, or Nagari) by the Minangkabau customary law community [22]. Ulayat land is an inheritance passed down from generation to generation, whose rights are with women, but as the holder of rights to Ulayat land is the head of the inheritance [22]. The customary fatwa states as follows:

“Berik tabang ka rice field (birds fly to the rice field), from Tabang ka yard rice field (from rice field flying to the yard), basuo on ground bato (meeting on the brick ground), from niniak down kamamak (from ninik down to Mamak), from Mamak turuk ka kamanakan (from mamak terun where), broken tumbuah lost baganti (broken growth lost changed) and pusako baitu juo (heirloom as well).”

The Minangkabau community had a sociocultural closeness to the land for centuries [23]. Customary land has a very important position for the Minangkabau community. Land is believed to be a source of livelihood, a symbol of identity determining social status, and one of the elements of matrilineal institutions. The position of customary land which is quite important is prone to disputes and conflicts both horizontally and vertically [17]. High heirloom assets in Minangkabau generally consist of property in the form of land. For the Minangkabau people, land is special which is a binding factor between members of society for the integrity of the people themselves. The legal relationship between community members and the common land gave birth to Ulayat rights on the land.

As is the case in Minangkabau, some countries in the world also show formal recognition of customary land rights, such as in Australia [24–26] and Canada. In the summary presented by [27], the formal recognition also raises various problems such as the interest in increasing the existence of indigenous people. On the other hand, [28] it also adds that formal recognition is also often the root cause of tensions with other nonindigenous local land users [28]. In other words, Ulayat rights, apart from being the hallmark of the Minang community as a form of local wisdom, are also complicated on the other hand because of the pattern of placing ethnicity above national identity and interests, such as the construction of road infrastructure for various public interests.

### 3.4. Public Perception and Attitude

Social impact is a consequence of a certain event or situation that results in changes in views or perceptions [29], changes in livelihoods or employment opportunities, patterns of social interaction, values, customs, and community culture. These changes can be positive or negative towards society. The construction of the East bypass Pasar Amor Ring road is predicted to have a positive impact on the surrounding community, but it does not rule out the possibility of various negative impacts that need to be anticipated. These various perceptions are used as a basis for identifying the positive and negative impacts of the construction of the East bypass Pasar Amor Ring road as shown in Table 3.

#### 3.4.1. Public Perception of the Socialization of the Road Development Plan

The socialization of the bypass Pasar Amor road development plan by the central, provincial, and district governments has been carried out to the community through traditional leaders, Ninikmamak, Nagari Customary Density, and the Nagari Consultative Body (Bamus). The bypass Pasar Amor road construction plan has also been disseminated to the public through these figures in informal meetings. The impact of this socialization is physically seen in the physical condition of most of the land included in the route of the bypass extension which is still empty even though there are few buildings. The results of the study found that the socialization carried out did not occur in a sustainable manner, so people began to doubt the certainty of the implementation of the bypass Pasar Amor road construction plan as the results of an interview with the chairman of Bamus Nagari Ladang Laweh:

“This is perhaps one of our thoughts that has been stuck. I often have meetings with members of the DPRD of Agam Regency and West Sumatra Province at the Mayor's office and subdistrict office and meetings at the Senayan feed. What I always ask is the continuation of the construction of Jalan bypass. That is the problem that I convey on behalf of the community. We question the extension of the bypass road to Pasar Amor, this is a form of our seriousness in supporting the extension of the bypass Pasar Amor” (Interview with Y, 2020).

Furthermore, it was conveyed that community leaders were willing to facilitate the socialization of the bypass road Pasar Amor road construction plan before the construction of the bypass road extension was carried out. The community expects the government to be able to inform the objectives of infrastructure development, the positive and negative impacts of infrastructure development on the community, land acquisition mechanisms, and compensation systems as well as land prices offered by the government. This information is very important as consideration for the community to provide support or approval to the road construction plan and the acquisition of the affected land. Furthermore, it was conveyed that the socialization should include landowners, tribal administrators (chairmen of 11 tribes), affected communities, Walijorong, Walinagari, KAN, BamusNagari, Pemuda, BundoKanduang, Niniakmamak, pious ulama, and Cadiak clever.

#### 3.4.2. Community Attitude towards Bypass Amor Road Development Plan

Communities in Banuhampu and Sungai Pua subdistricts generally support the bypass Amor road construction plan because in addition to overcoming congestion, road construction can facilitate access to Amor market and encourage the emergence of new economic growth centers such as the growth of shop houses or rental houses on the side of the road. This was conveyed by the District Apparatus as follows:

“...Because our efforts to go to the outer fields have not found a solution, so for the by-pass we actually in the subdistrict at that time had close meetings with community leaders and mayors. If the Nagari which is directly adjacent to Bukittinggi is the guardian of 4 tribes. By WalnagAmpek, tribes and community leaders in principle agreed to bypass. After Taluak 4 tribes then pass NaagariLaweh fields. In the Nagari, the field of Laweh together with the community leaders agreed in principle for land acquisition but had to depend more on the land owner. If we are community leaders to strengthen it, it is possible but to force us we can't afford it, so it has been brought together with Toma and Walnag. Then from the direction of Bukittinggi, namely NagariCingkariang. NagariCingkariang agrees in principle but land acquisition depends on the land owner. Therefore there is more socialization. In fact, we invited the Pua River. Instead, the community wants this by-pass road to go through to Jalan Koto Baru, not the original concept, through Cingkariang (so dicingkaring does avoid a little in the field outside than in Cingkaring there will be more traffic jams. Because during Lebaran, traffic jams can reach Cingkariang and Sei).Buluah. If the by-pass passes in Cingkariang, there is no solution, so it is recommended to go to the new Koto near the Amor market.” (Interview of the SeiPua Sub-District Head, 2020).

The results of the interview show that government officials, Nagari officials, and community leaders support the construction of the bypass road because they are optimistic about the positive impact of road construction on the progress of Nagari development. The following are the results of the researcher's interview with the Banuhampu subdistrict head:

“There is SeiPua, Sariak and the last Batagak, NagariBatuPalano, which comes out in Koto Baru or near the Amor Market. So after we had a meeting there were some people with Toma. We plan to do a tracking bypass for the old and pioneering areas that can be passed, but at that time, because of Toma who was involved in land acquisition in the past, many obstacles were eventually delayed, however, after coordinating with the Perkim at that time, we did not have any technical personnel in the subdistrict. If you don't have technical personnel for tracking, just go the usual way, sir. Of course, we want tracking along the possible paths that are suitable to be passed. If you follow the old path, there are a few obstacles in the Laweh field, the road that goes through from Prapatan Padang Luar from here goes straight there. If this is the old route, then there is a building. So you have to find a new route. That's the result of our efforts to continue the development of by pass. So when we collide, we invite PU but it doesn't happen. “ (Interview with the Banuhampu subdistrict head, 2020).2

#### 3.4.3. Community Perception of Land Acquisition

The control and management of Ulayat land is intended to protect and maintain the life and existence of the community (cultural existence). In addition, Ulayat land also contains elements of religion, history, and even magical elements and aims to prosper the people in it [30]. The high position of Ulayat land in the life of the community becomes an obstacle in land acquisition for road construction because it requires the agreement of all members of the communal group, whether ethnicity, clan, or Nagari. Therefore, in the land acquisition process, approaches that can be accepted by the community are needed so as not to cause rejection from the community which ultimately leads to conflicts both vertically and horizontally [31].

The process of acquiring communal land for the construction of Jalan Bypass Amor is also not easy because it is communal land owned communally by tribes, clans, and Nagari, so the process of releasing it also requires mutual approval from clan members, most of whom live overseas, according to an interview with one of the leaders. Public:

“... So, Katiko, we want to release the rice fields for a by-pass, or not immediately, Mr. DapekBarangnyodoh, we have to first ask for the approval of the immigrants sporadically. We buy and sell the price is Rp. 2,000,000 per meter, you can't do it right away. We have to seek mutual agreement between us, the land owners, and ampekranjikaateh. Grandma, mother ambo and son ambo. Ambo's children as heirs of the heirs are okay if men don't enter. Men have no right to land, madam, only manjago, who has the right to bundokanduang. The land products can be brought to the house of Ambo's wife.” (Interview with Y, 2020).

Furthermore, these approaches are not only carried out to the community but also to ninikmamak, tribal leaders and GantoBenoa who are niniakmamak associations and final decision makers in decision making in the Banuhampu and Sungai Pua subdistricts (BamusLadangLaweh).

The problem of land acquisition at the beginning of the bypass road construction was different from today. At the beginning of the construction of the bypass road, the land affected by the road construction was in the form of rice fields, but now the government will face more complex problems because the land affected by the extension of the bypass road has been built for shop houses or housing. This condition is as illustrated by the results of interviews with the Sungai Pua subdistrict head:

“After trying several meetings and conducting field surveys, there are indeed a lot of problems regarding land, agriculture, buildings and so on, plus all lands cannot be certified if they build it, if it's a ponyo, if it's a nephew, please even if it's not certified. It's quite heavy but for us, there is no other solution, the desert remains like that there are options such as flying over but back to the land problem. But as strong as the community is, the government is stronger. In the future, it is impossible for this to be allowed to continue like that, we demand development and smooth transportation and there is still a chance for the plan, many people who are affected have indeed refused.” (Interview with SeiPua Sub-district Head, 2020).

The community expects land acquisition to be carried out not with a consolidation system but with a buying and selling system because land acquisition with a consolidation system is considered very detrimental to the community. Land consolidation is a development method which is one of the policies for regulating land tenure, adjusting land use with land use/spatial planning, and land acquisition for development purposes as well as environmental quality/maintenance of natural resources [32]. The community refused consolidation because they did not receive proper compensation. The rejection of the consolidation system is also based on experience during the initial land acquisition of Jalan bypass. The conflict between the community and the government led to a trial and had dragged several government officials into it.

#### 3.4.4. Perception of Compensation

Compensation is compensation given to parties affected by road construction in the form of money, replacement land, and resettlement or other forms based on the agreement of both parties [33, 34]. The form of compensation is given according to Article 18 of Law Number 2 of 2012 concerning that land procurement can be in the form of money, replacement land, resettlement, shared ownership, or other forms agreed upon by both parties. The value of compensation is obtained based on the agreement between the entitled party and the implementer of the procurement or court decision that has obtained legal force [34].

Compensation in the context of this research is compensation in the form of money. The arrangement of compensation in the form of money is regulated in Presidential Regulation No. 71 of 2012, and its technical guidelines are regulated in the Regulation of the Head of BPN No. 5 of 2012. The compensation desired by the community who owns the land is compensated by a buying and selling system based on market prices and rejecting compensation using a consolidated system. The results of an interview with the Chairman of the Bamus Nagari Ladang Laweh follows:

“So the core issue of the by-pass problem is that if the by-pass becomes a connection between Taluak and Amor with a consolidation system, it is certain that the community is not willing. But if there will be an extension of the by-pass with a buying and selling system instead of compensation, the community will accept it.” (Interview with Y, 2020).

The selling price of land around the research site is quite high, ranging from Rp. 1.000.000 to Rp. 4,000,000, depending on the distance from the road and the interpretation of the economic value generated by the land. Based on the results of interviews conducted with community leaders, it was concluded that there were several reasons given by the community: first, consolidation was considered detrimental to the community and opened up opportunities for other parties to take advantage of the process as experienced in the initial construction of the bypass road; second, the status of land ownership is customary land which should not be traded; third, the affected land is productive land which is mostly planted with horticultural crops of high economic value; and fourth, agricultural land is the main source of livelihood for the majority of the population.

Furthermore, the proceeds from the sale of the high-rise Pusako land have not been lost but have changed its function, which was originally a land or rice field, now into a shop house that can be rented out. This was conveyed by the Deputy of Bamus Ladang Laweh:

“Now it's the turn of the high-rise Pusako, which will later be converted into shop houses and the ko-ruko shop will remain as the high-rise Pusako. Usually, the rice fields are bought and rented out, now the shophouses are rented out. Oh yo, just pouring the time on that. So we don't refuse, sir, but only support Caronyo.” (Interview with Ahda, 2020).

Another opinion was conveyed by one of the community leaders of Ladang Laweh. The decision to sell Ulayat land for road construction was based on two considerations, namely: first, which stated that the proceeds from the sale of land from the compensation for the road construction were used to build shop houses or convert existing land to build shop houses or tenement houses that could be rented out, secondly, it would still maintain the rice fields because most of the members of his people live in the overseas.

### 3.5. Impact and Mitigation Strategies

The construction of the East Ring Road bypass Pasar Amor will have an impact on the community from the economic, environmental, and social aspects. The social impacts identified in this study are divided into two categories, namely, positive impacts and negative impacts.

The positive impact obtained at the road construction stage is the recruitment of workers from the surrounding area so that it can provide new jobs and reduce unemployment. In addition, the selling price of land in the area around the new road has increased. Furthermore, the positive impact obtained after the road construction process is complete is the reduction of congestion in the Padang Luar area and the opening of road access to the Amor market. The opening of road access to Pasar Amor is expected to spur new economic growth and settlements in the area along the new road. The community has other business opportunities besides farming, such as trading and being the owner of a shop or house for rent. The center of new economic growth is an attraction for immigrants to work in that place.

While the negative impact that occurs in the construction process is the emergence of land speculators to the detriment of the community and government, and the emergence of air and noise pollution around the road construction area. The negative impact after road construction are, namely, changes in lifestyle and the disappearance of traditional values. The construction of the bypass Pasar Amor road allows for interaction between the natives and the immigrants. This interaction can change people's lifestyles which are feared to cause the loss of cultural and customary values that are upheld by the Nagari community. The fading of cultural customary values has also resulted in the weakening and shifting of the role of Mamak, the waning of the morals and morals of the younger generation from the philosophy of life of the Minangkabau people, namely, Adat Basandi Syara', Syara' Basandi Kitabullah, and other noble values passed down from generation to generation such as mutual cooperation, deliberation, and other noble values. In addition, the interactions that occur are feared to cause social conflicts between indigenous people and immigrants due to differences in welfare levels and lifestyles.

The increasing selling price of land and the diminishing role of the Mamak as guardians of communal land resulted in a shift in land ownership from communal ownership to private property. The shift in land ownership has resulted in massive changes in the function of agricultural land to nonagriculture in the two research areas. This condition was not expected by the Ninik Mamak, who made an agreement in 1980 that land in Banuhampu and Sungai Pua should not be sold to migrants until now. The positive and negative social impacts of the bypass Amor road extension can be seen in Table 3.

Henceforth, a strategy for mitigating the social impacts of the bypass Amor road construction needs to be prepared to prevent adverse impacts on the community as well as the bypass Amor road construction project. This social impact mitigation strategy is prepared by incorporating the values of local wisdom that grows and develops in the Minangkabau community. Based on the results of the analysis of secondary and primary data, a mitigation strategy is formulated as shown in Table 4.

## 4. Conclusion

In planning the construction of alternative roads, it is necessary to identify problems and impacts that arise during the preconstruction, construction, and postconstruction periods. The positive impact expected from the construction of this road is to overcome the main problem, which is to overcome congestion, and the associated impact after the construction of a new road is to create new economic sources due to the opening of the accessibility of an area. However, it is undeniable that the negative impacts of the construction of this road such as the emergence of land speculators, horizontal and vertical conflicts, the transfer of customary land into property rights, and lifestyle changes due to associating with immigrants.

Land acquisition is a major obstacle in infrastructure development. Land for the Minangkabau community is a source of livelihood, a symbol of identity that determines social status, and one of the elements of matrilineal systems. Therefore, it often causes conflicts in the process of liberation. So, it is necessary to mitigate negative social impacts based on local wisdom with a sociocultural approach continuously to prevent conflict and resistance.

To formulate social impact mitigation, so that the main attention is needed in assessing community support, among others: first, Ulayat land as a customary symbol of the Minang community must be protected and its existence must be protected through the approval mechanism of the people and mamak in the release of land; second, the public interest is the foundation of the principle in efforts to mitigate, negotiate, and provide compensation for road infrastructure development; third, development, especially in readiness for job transfer and land commodity transfer, must be designed to anticipate local social, economic and cultural changes affected by road construction.

Mitigation efforts that must be carried out continuously at every stage of the bypass Amor Road construction include the following: first, socialization and transparency, this is very important to prevent conflicts and resistance from the community. The community expects the government to inform the objectives of infrastructure development, positive and negative impacts, land acquisition mechanisms, and compensation systems as well as land prices offered by the government. Especially for land acquisition issues, expected land acquisition does not harm the community, so it will reduce the possibility of conflict occurrence. The government must be ready with the financial capacity for land compensation, given the high economic value of land in this area. This information is very important as a consideration for the community to provide support or approval to the road construction plan and the acquisition of the affected land. Second, local governments are required to prepare regulations, strategies, and policies to provide new jobs for people affected by road construction. People who originally worked on land which later turned into roads will lose their source of income; third, the community is involved in the deliberation process to listen to each other's opinions and wishes regarding the form of loss so that an agreement occurs between the owner of land rights and those who need the land; fourth, it opens the opportunity to enter into a cooperation agreement for the use of Ulayat land for development, Ulayat land is considered as capital participation on the condition that the land is not transferred, not traded, and ownership is not changed.

This mitigation effort should be carried out in parallel with the approach to the community through traditional leaders (Ninik Mamak), and influential minang immigrant ties (Perantau), cleric (Alim Ulama) and professional local (Cadiak Pandai). The approach taken is not only formal but also informal, such as discussing with sitting in the lapau (coffee shop) which will have more impact. Furthermore, involving the local community as a work team during preconstruction and postconstruction will be more efficient and effective to build trust and guarantee. Ulayat land is a customary symbol of the Minang community, and its existence must be protected so that in releasing land for road construction it must go through the Ninik Mamak approval mechanism as tribal chief and the community as members of the tribe. Furthermore, it prepares the community to face changes after road construction, such as job transfers, agricultural land commodity transfers as well as changes in cultural and social relations.

The weakness of this research is that the social impact assessment is an estimate, so the availability of accurate data is very important for the accuracy of the impact assessment that is only felt in the medium or long term. Meanwhile, from the scope of our study, we only focus on identifying the impacts and mitigating the construction of the bypass Amor road only from the social aspect, thus ignoring other aspects that are also important to anticipate. In the future, the scope of the study can be expanded to other aspects. In addition, our study has not considered the element of sustainability as an important topic of sustainable development.

## Figures and Tables

**Table 1:** Details of areas affected by the construction of infrastructure roads.

District-nagari	An area	Number of jorong	Total population	Population density (Life/Km^2^)	Area potential
Banuhampu	28.45	43	42241	1,484.75	Agriculture, trade, and services
LadangLaweh	4.42	4	6537	1,478.96	
Cingkariang	5.07	6	5442	1073.37	
Padang Lua	3.42	3	7428	2,171.93	

Sungai Pua	44.29	27	24947	563.26	Agriculture, trade, weaving, and services
BatuPalano	3.47	5	3050	878,96	
Batagak	9.84	7	3624	368,29	
Sariak	11.4	7	2170	190.35	
Sungai Pua	16.9	5	12371	732.01	

Source. Sungai Pua District in Figures 2020 and Banuhampu District in Figures 2020

**Table 2:** Study area directly affected by East Ring road construction on bypass Amor.

Road construction position	Districts	Nagari
Stage I	Banuhampu	Taluak
		Padang Laweh

Stage II	Banuhampu	Padang Laweh
	Sungai Pua	Cingkariang
		Sungai Pua

Stage III	Sungai Pua	Sariak
		BatuPalano

Source. Tracing Results (2020).

**Table 3:** Identification of the social impacts of bypass road Pasar Amor construction.

No.	Social impact criteria	Identification of social impact
1	Positive	(i) Reduce congestion (ii) Easy access and mobility (iii) Create jobs and reduce unemployment (iv) Unlock new settlements

2.	Negative	(i) Land speculators appear (ii) Community resistance to land acquisition and compensation (iii) Horizontal conflict arises between the people who are pro and contra of road construction (iv) Vertical conflict arises due to dissatisfaction with the land acquisition and compensation process (v) Social unrest in the community as a result of the conflict (vi) Livelihood change occurs as the population who originally farmed as a hereditary livelihood changed to the service and trade sectors (vii) Shifting customary land into property rights (viii) Changes in lifestyle with the disappearance of customs that are upheld, especially in the younger generation due to mixing with immigrants

Source: interview results, 2020.

**Table 4:** Social impact mitigation strategy bypass road construction of Amor.

No.	Project/activity stages	Negative social impact	Mitigation strategy	Person responsible
1.	Preconstruction	Community resistance to land acquisition and compensation	Conducting intensive and responsive socialization and approach	District government, camat, Wali Nagari, community leaders, and traditional leaders
		Horizontal conflict pros and cons appears	Community engagement approach through community-based approach	District government, camat, Wali Nagari, community leaders, and traditional leaders
		Horizontal conflicts arise due to dissatisfaction with land compensation	Improving socialization and communication as well as approaches to community leaders and traditional leaders	District government, camat, Wali Nagari, community leaders, and traditional leaders
		Social unrest arises due to conflict	Community engagement approach through community-based approach	District government, camat, Wali Nagari, community leaders, and traditional leaders

2.	Construction	The emergence of anxiety community due to air and noise pollution due to equipment mobilization and materials during construction.	(i) Covering the body of the vehicle carrying the material tightly using a tarpaulin so that no material is scattered on the road which has the potential to increase the dust content (ii) The speed of the transport vehicle used by the activity implementer (contractor) is not more than 20.0 km per hour (iii) Clean the soil material that sticks to the tires when leaving the activity location so that it does not pollute the road which has the potential to increase the dust content (iv) Using transportation vehicles that are still in good condition (mechanical availability > 80%) so that they do not cause high noise (v) Limit the number of vehicles used at the same time (vi) Maintaining vegetation in the area adjacent to the nearest residential location to reduce noise levels	Implementing contractor, supervised by KLH Kota Bukittinggi and BPLH Kab. Agam, Bukittinggi city health office and kab religion

3.	Postconstruction	Livelihood change	Prepare alternative livelihoods based on community interest, local potential, market opportunities, and financial feasibility and provide capital for business	District government subdistrict head Wali Nagari
		Shifting of communal land to own	Increasing the role of Ninik Mamak as guardians of Ulayat property	District government, camat, Wali Nagari, community leaders, and traditional leaders
		There is a change in lifestyle due to the erosion of traditional and cultural values	Promoting the malakok tradition of tribes and Mangaku Mamak for immigrants	Community leaders and traditional leaders

Source. Research Results (2020).

## Data Availability

Data is taken from Institution, namely, Nagari or Village, Central Bureau of Statistics of West Sumatra Province, Interviews with Heads of Nagari or Village Heads, Nagari or Village Consultative Body, Interviews with Sub-Heads of District.
